# Prognostic accuracy of early warning scores for predicting serious illness and in-hospital mortality in patients with COVID-19

**DOI:** 10.1371/journal.pgph.0002438

**Published:** 2024-03-28

**Authors:** Mehnaz Kamal, S. M. Tafsir Hasan, Monira Sarmin, Subhasish Das, Lubaba Shahrin, A. S. G. Faruque, Mohammod Jobayer Chisti, Tahmeed Ahmed

**Affiliations:** 1 Nutrition and Clinical Services Division, International Centre for Diarrhoeal Disease Research, Bangladesh (icddr,b), Dhaka, Bangladesh; 2 Office of Executive Director, International Centre for Diarrhoeal Disease Research, Bangladesh (icddr,b), Dhaka, Bangladesh; Department of Public Health and Allied Sciences, and Department of Anthropology PAKISTAN

## Abstract

A simple bedside triage tool is essential to stratify COVID-19 patients in the emergency department (ED). This study aimed to identify an early warning score (EWS) that could best predict the clinical outcomes in COVID-19 patients. Data were obtained from medical records of 219 laboratory-confirmed COVID-19 positive patients. We calculated 13 EWSs based on the admission characteristics of the patients. Receiver operating characteristic (ROC) curve analysis was used to assess the performance of the scores in predicting serious illness and in-hospital mortality. The median patient age was 51 (38, 60) years, and 25 (11.4%) patients died. Among patients admitted with mild to moderate illness (n = 175), 61 (34.9%) developed serious illness. Modified National Early Warning Score (m-NEWS) (AUROC 0.766; 95% CI: 0.693, 0.839) and Rapid Emergency Medicine Score (REMS) (AUROC 0.890; 95% CI: 0.818, 0.962) demonstrated the highest AUROC point estimates in predicting serious illness and in-hospital mortality, respectively. Both m-NEWS and REMS demonstrated good accuracy in predicting both the outcomes. However, no significant difference was found between m-NEWS (*p* = 0.983) and REMS (*p* = 0.428) as well as some other EWSs regarding the AUROCs in predicting serious illness and in-hospital mortality. We propose m-NEWS could be used as a triage score to identify COVID-19 patients at risk of disease progression and death especially in resource-poor settings because it has been explicitly developed for risk stratification of COVID-19 patients in some countries like China and Italy. However, this tool needs to be validated by further large-scale prospective studies.

## Introduction

The sudden emergence of novel coronavirus disease (COVID-19), an infectious disease caused by the severe acute respiratory syndrome coronavirus 2 (SARS-CoV-2), has quickly become a global threat. In December 2019, it was discovered in Wuhan city in central China and rapidly spread throughout China and other regions of the world. Globally, 279,114,972 confirmed cases of COVID-19 were found including 5,397,580 deaths till 22 December 2021 [1]. A study from Wuhan reported that the mortality rate among critically ill COVID-19 patients was 62% and 81% in patients who required mechanical ventilation [2], whereas the mortality rate among the population in India was 10.5% [3]. Bangladesh is also facing an overwhelming outbreak of this disease since March 8, 2020 [4]. This pandemic posed a huge challenge to the healthcare system of Bangladesh. The increasing number of patients with COVID-19 has created a burden on all tertiary level facilities [5]. In this complex situation, it became mandatory to develop systematic clinical strategies for efficient use of medical efforts to effectively treat COVID-19 and most importantly, identify a sub-population requiring immediate critical care to reduce fatal consequences [5, 6].

Recent findings from Wuhan reported that having certain co-morbidities such as hypertension (HTN), diabetes mellitus (DM), acute kidney injury (AKI), coronary artery disease (CAD), acute hepatic injury, elevated c-reactive protein (CRP), elevated d-dimers, interleukin-6 (IL-6), lymphocyte count, and procalcitonin levels were associated with poor outcome and hospital mortality [7]. A person with COVID-19 may be asymptomatic or have mild symptoms like cough, fatigue, fever, and sore throat, but it can lead to life-threatening complications in severe cases like those with acute respiratory distress syndrome (ARDS), coagulopathy, and severe sepsis [8]. At present, a critical value of biomarkers such as WBC count, CRP, pro-calcitonin, and d-dimers are widely used to stratify the disease severity in COVID-19 patients, but they often end up proving non-significant, expensive, and takes longer time for initiating imperative measures [9]. Due to limited resources, recognizing high-risk patients at an early stage by using simple physiological parameters may reduce the number of severe cases [10]. Amid the pandemic, to maximize the use of existing resources and minimize the use of expensive laboratory investigations, standardized clinical scoring systems are critically important to identify COVID-19 patients who require immediate attention that may help clinicians in complex decision-making scenarios [5].

The Early Warning Scores (EWSs) are a variety of physiologic scoring systems which are based on bedside indices like respiratory rate, heart rate, blood pressure, oxygen saturation (SpO_2_) and consciousness level that can be obtained easily for early recognition of deteriorating patients or those who are vulnerable for potential deterioration and for intervening with appropriate measures on time [11]. It was first developed in James Paget University Hospital, Norfolk, UK and was presented at a conference of the Intensive Care Society in late 1990’s [12]. Several EWSs are routinely used in developed countries [13–15]. Amid this pandemic, it has been hypothesized that a readily available, understandable EWS, which can best predict high-risk cases instantly, can be a powerful tool [16]. In the present study, we have compared the accuracy of 13 established early warning scores (EWSs), calculated based on admission characteristics, in predicting the development of serious illness and death among COVID-19 positive patients.

## Material and methods

### Study site

This study was conducted in the Dhaka Hospital of International Centre for Diarrhoeal Disease Research, Bangladesh (icddr,b). Dhaka Hospital has inpatient and outpatient facilities for diarrhoeal and other related childhood illnesses. Each year it treats approximately 150,000 patients with diarrhoeal illnesses [17]. People from socioeconomically disadvantaged communities are the majority of patients visiting the hospital and reside in urban or peri-urban Dhaka [18]. In order to safeguard the health of its employees during this pandemic, the organization gradually increased the range of services it offered, starting with basic COVID-19 screening, triaging, and management of severe cases. It also established one inpatient facility equipped with the tools needed to treat critical cases of COVID-19.

### Study design

This is a single-center, retrospective analysis of data obtained from COVID-19 cases managed at Dhaka Hospital, icddr,b. We reviewed the clinical record of all adult COVID-19 positive patients consecutively admitted to our emergency department in a one-year period of time (April 15, 2020 to April 14, 2021). ‘National guidelines on clinical management of coronavirus disease’ were followed for identification, clinical classification, and treatment plan.

### Study population and data source

The study population comprised icddr,b staff, and their family members who were COVID-19 positive from April 15, 2020 to April 14, 2021 and whose diagnosis was confirmed with a real-time reverse-transcriptase polymerase-chain-reaction (RT-PCR) assay of nasal and/or pharyngeal swab specimens. Data were accessed for research purposes in early June 2021 following the approval of the study by the Ethical Review Committee.

**Fig 1:**
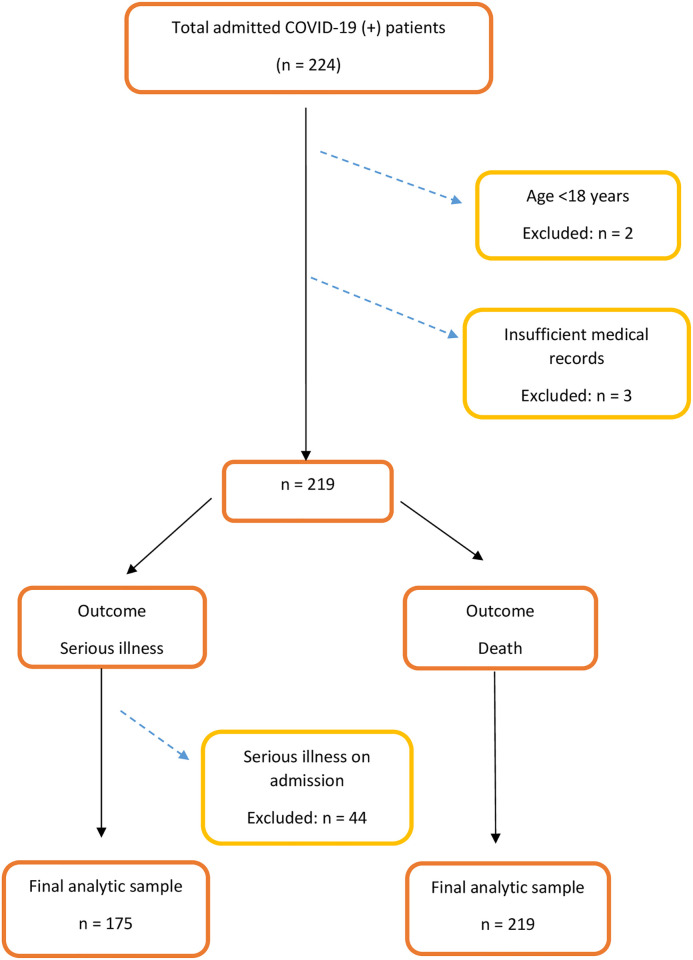
Flowchart showing selection of study population.

Patients who were 18 years old and above during admission and those with complete medical records were included in this study. Of the 224 cases that met the inclusion criteria, we excluded 2 for bearing age below 18 years and 3 cases for incomplete medical records. We excluded 44 patients who had a serious illness on admission. For this, a total of 175 COVID-19 positive patients were analyzed to predict the development of serious illness. However, a total of 219 COVID-19 positive patients were analyzed in this study for predicting in-hospital mortality by using EWSs (Fig 1).

Relevant data were extracted from the patients’ medical records. We abstracted data on patient attributes, including age, sex, respiratory rate, heart rate/pulse, temperature, oxygen saturation by pulse oximetry, supplemental oxygen, blood pressure, Glasgow coma scale (GCS) score, alertness, response to voice and pain, unresponsive (AVPU) scale and history of co-morbidities on admission. All data of COVID-19 patients were kept as hard copies in hospital medical records.

### Selection of scoring system

After an extensive literature review, we have selected 16 EWSs that matched the objective of our study. We took those EWSs which have been used in the emergency department or similar settings and are using vital sign parameters to formulate a numerical score that can predict clinical outcomes on admission [14]. Among the 16 EWSs, we excluded three; Modified Early Warning Score with GCS (MEWS-GCS) [19] and Abbreviated VitalPac Early Warning Score (AbViEWS) [20] because of having similar components as Modified Early Warning Score (MEWS) and VitalPac Early Warning Score (ViEWS) and in case of National Early Warning Score (NEWS) [21], we kept National Early Warning Score 2 (NEWS2) as it has already been recommended by the Royal College of Physicians in diagnosing deteriorating COVID-19 patients [22]. Total 13 EWSs including National Early Warning Score 2 (NEWS2) [23], Modified National Early Warning Score (m-NEWS/ NEWS-C) [24], Quick Sepsis-related Organ Failure Assessment (qSOFA) [25], Rapid Acute Physiology Score (RAPS) [26], Modified Early Warning Score (MEWS) [27], Standardized Early Warning Score (SEWS) [28], Rapid Emergency Medicine Score (REMS) [29], Goodacre Score [30], Worthing Physiological Score (WPS) [31], Hamilton Early Warning Score (HEWS) [32], Groarke Score [33], VitalPac Early Warning Score (ViEWS) [16] and Confusion Respiratory Rate Blood Pressure Age 65 score (CRB-65) [34] were evaluated for formulating predicting outcome in our study based on the clinical parameters on admission day.

All the scores had similar variables, such as respiratory rate, heart rate, temperature, blood pressure, GCS and AVPU scale, SpO_2,_ and the need for supplemental O_2._ We divided our 13 EWSs into three categories on the basis of supplemental O_2_ requirement and presence of SpO_2_ variable in the scoring system. The first category consists of those EWSs that do not require SpO_2_ or supplemental O_2_ to calculate the total score, such as MEWS, RAPS, Goodacre, CRB-65, and qSOFA. Another category of EWSs was who consider SpO_2_ but not the requirement of supplemental O_2_ to calculate the total score for instance; SEWS, REMS, Groarke, and WPS. The last group was those EWSs that have both SpO_2_ and the requirement of supplemental O_2_ variable to calculate the total score; NEWS2, m-NEWS, HEWS, and ViEWS. A full description of all the categories of EWSs and their scoring charts has been given in S1 and S2 Tables.

### Outcomes

The study outcomes were the development of serious illness within 7 days of admission and in-hospital mortality within 28 days from admission. As per the national guideline on clinical management of coronavirus disease 2019, we defined serious illness as the clinical syndromes included in severe and critical stages, such as severe pneumonia, sepsis, ARDS, and septic shock. Severe COVID-19’ was defined as a patient with fever or suspected respiratory infection plus one of these signs: respiratory rate > 30 breaths/min, severe respiratory distress; or SpO_2_ ≤ 93% on room air along with severe pneumonia and sepsis. ‘Critical COVID-19’ was when a patient had severe COVID-19 with septic shock and ARDS [35].

### Ethics statement

The study (PR-21025) was reviewed and approved by the Institutional Review Board (Research Review Committee and Ethical Review Committee) of icddr,b. As it was a secondary data analysis of clinical care-related data, no interview of any patient was done. Hence, no consent was required for this study.

### Statistical analysis

Data were analyzed using Stata/PC (StataCorp, College Station, Texas 77845 USA, version 15.1). Continuous variables were presented as median, 25% quartile and 75% quartile. Categorical variables were presented using frequencies and percentages. Data were compared using the Mann-Whitney U test for continuous variables and the Chi-square test or Fisher’s exact test for categorical variables. Receiver operating characteristic (ROC) curve analysis was used to estimate the performance of the EWSs in predicting the development of serious illness and in-hospital mortality. Nonparametric ROC curve analyses were performed using the *roctab* command in Stata. *roctab* command provides the estimation of the area under the ROC curve (AUROC) and its 95% confidence interval (95% CI) by using an algorithm suggested by DeLong, DeLong, and Clarke-Pearson and asymptotic normal confidence intervals [36]. *roctab* command was also used to compute the sensitivity, specificity, and accuracy (% correctly classified) for all possible cut points. The Youden index was used to estimate the optimal cut-off point. The Youden method maximizes the sum of sensitivity and specificity [37]. Pairwise comparisons between ROC curves of different EWSs in predicting the development of serious illness and in-hospital mortality were made using the *roccomp* command in Stata. *roccomp* provides a test for the equality of the AUROCs, using an algorithm suggested by DeLong, DeLong, and Clarke-Pearson [36]. A *p*-value of less than 0.05 was considered statistically significant.

## Results

### Patient characteristics

The baseline characteristics of our participants are shown in Table 1. Of the 219 patients, the median age was 51 (IQR 38, 60) years, 59.4% were males, and 25 (11.4%) died. In our sample, all the patients who died developed respiratory failure. However, a few of them also had other complications during the course of their illness, including heart failure and septic shock.

**Table 1:** Admission characteristics of COVID-19 positive patients admitted to icddr,b Dhaka Hospital.

Characteristics	All Patients (n = 219)	Serious Illness^a^		*p* value	Death		*p* value
		No 114 (65.1)	Yes 61 (34.9)		No 194 (88.6)	Yes 25 (11.4)	
Age, years	51 (38, 60)	42 (34, 53)	56 (47, 65)	<0.001	49 (37, 58)	65 (60, 72)	<0.001
Sex, male	130 (59.4)	71 (62.3)	35 (57.4)	0.527	115 (59.3)	15 (60)	0.945
HR, beats/min	90 (80, 100)	88 (80, 98)	92 (81, 100)	0.170	90 (80, 100)	95 (84, 110)	0.177
RR, breaths/min	24 (20, 32)	20 (18, 26)	28 (24, 34)	<0.001	24 (20, 30)	36 (30, 42)	<0.001
SpO_2,_ %	97 (95, 98)	98 (97, 99)	97 (96, 98)	<0.001	98 (96, 98)	91 (74, 95)	<0.001
Supplemental O_2_	40 (18.3)	0 (0)	0 (0)	-	23 (11.9)	17 (68)	<0.001
SBP, mmHg	121 (110, 135)	122 (110, 133)	120 (110, 132)	0.680	120 (110, 132)	127 (114, 145)	0.094
DBP, mmHg	80 (70, 87)	82 (74, 90)	76 (70, 84)	0.004	80 (70, 87)	75 (64, 87)	0.253
MAP, mmHg	92.6 (84, 102)	96.6 (87, 102.6)	91.6 (83.3, 100.6)	0.082	92.6 (85, 101.3)	91.6 (80, 108.3)	0.888
Temperature, °C	36.4 (36, 37)	36.2 (36, 36.9)	36.4 (36, 37.2)	0.678	36.3 (36, 37)	37.0 (36.4, 37.6)	0.009
GCS	15 (15, 15)	15 (15, 15)	15 (15, 15)	-	15 (15, 15)	15 (15, 15)	<0.001
**Lab Parameters**							
^1^Hb, g/dl	12.2 (11.1, 13.4)	12.7 (11.4, 13.9)	12.1 (11.1, 13.2)	0.096	12.2 (11.1, 13.4)	12.1 (11.1, 13.7)	0.923
^2^TLC, 10^9^/L	6.83 (5.42, 9.06)	6.65 (5.12, 8.48)	6.18 (5.32, 8.01)	0.998	6.715 (5.28, 8.48)	9.28 (6.21, 11.8)	0.002
^3^Neutrophil, %	69.4 (60.6, 78.6)	62.2 (57.6, 71.7)	69.5 (62.8, 76.5)	0.002	66.2 (58.9, 76)	83.3 (75.8, 87.4)	<0.001
^4^Lymphocyte, %	23.5 (15.2, 31.5)	29.3(20.5, 35.2)	23.1(15, 30.6)	0.002	25.4 (18.2, 32.8)	11.2 (6.8, 15.5)	<0.001
^5^S. Sodium, mmol/L	133 (130, 135.5)	134.3 (131, 137.3)	133 (129.4, 135.2)	0.068	134 (130, 136)	131 (129, 134)	0.021
^6^S. Potassium, mmol/L	3.78 (3.4, 4.1)	3.62 (3.27, 3.92)	3.71 (3.4, 3.98)	0.370	3.72 (3.35, 4.03)	3.96 (3.58, 4.46)	0.015
^7^S. Creatinine, μmol/L	79.8 (63, 97.7)	68.8 (61.1, 86.1)	81.9 (66.7, 105.6)	0.026	73.03 (60.85, 92.25)	97.7 (79.9, 120.5)	0.001
^8^D-dimer, pg/ml	507 (294, 858.3)	409.5 (285.6, 656.5)	446.5 (272, 855)	0.466	446.6 (294, 786)	755.4 (587.5, 1754)	0.003
^9^CRP, mg/dl	4.03 (1.19, 12.7)	1.49 (0.35, 5.26)	4.09 (1.47, 9.83)	0.004	3.55 (1.15, 10.02)	15.5 (1.22, 24.7)	0.008
**Comorbidities**							
HTN	98 (44.7)	37 (32.5)	36 (59)	<0.001	83 (42.8)	15 (60)	0.103
CAD	34 (15.5)	7 (6.1)	17 (27.9)	<0.001	24 (12.4)	10 (40)	0.001
COPD	35 (16)	18 (15.8)	14 (22.9)	0.243	32 (16.5)	3 (12)	0.774
CKD	5 (2.3)	0 (0)	2 (3.3)	0.120	0 (0)	5 (20)	<0.001
DM	77 (35.2)	31 (27.2)	25 (41)	0.062	64 (33)	13 (52)	0.061
Thyroid disease	17 (7.8)	7 (6.1)	5 (8.2)	0.755	13 (6.7)	4 (16)	0.112
CNS disease	12 (5.5)	3 (2.6)	4 (6.7)	0.240	7 (3.6)	5 (20)	0.006
**Early Warning Scores (EWSs)**							
**EWSs that do not consider SpO**_**2**_ **or requirement of supplemental O**_**2**_ **to calculate the total score**							
MEWS	2 (1, 3)	2 (1, 2)	3 (2, 4)	<0.001	2 (1, 3)	3 (3, 4)	<0.001
RAPS	1 (0, 3)	0 (0, 1)	1 (0, 3)	<0.001	1 (0, 2)	3 (2, 3)	<0.001
Goodacre	3 (1, 5)	2.5 (0, 4)	3 (3, 6)	<0.001	3 (1, 5)	5 (3, 8)	<0.001
CRB-65	0 (0, 1)	0 (0, 0)	1 (0, 1)	<0.001	0 (0, 1)	1 (1, 2)	<0.001
qSOFA	1 (0, 1)	1 (1, 1)	1 (1, 1)	<0.001	1 (0, 1)	1 (1, 1)	0.003
**EWSs that consider SpO**_**2**_ **but not requirement of supplemental O**_**2**_ **to calculate the total score**							
SEWS	2 (1, 4)	1 (0, 2)	2 (1, 3)	<0.001	1.5 (1, 3)	4 (3, 7)	<0.001
REMS	3 (1, 6)	2 (0, 3)	5 (3, 6)	<0.001	3 (1, 5)	9 (6, 12)	<0.001
Groarke	2 (1, 4)	1 (0, 2)	3 (1, 4)	<0.001	2 (1, 4)	6 (4, 7)	<0.001
WPS	2 (1, 4)	2 (0, 2)	2 (2, 3)	<0.001	2 (1, 3)	5 (3, 6)	<0.001
**EWSs that consider both SpO**_**2**_ **and requirement of supplemental O**_**2**_ **to calculate the total score**							
NEWS2	4 (2, 6)	2 (1, 4)	4 (3, 5)	<0.001	3.5 (2, 5)	9 (5, 10)	<0.001
m-NEWS	4 (2, 7)	2 (1, 4)	5 (4, 7)	<0.001	4 (2, 6)	10 (7, 12)	<0.001
HEWS	3 (1, 5)	2 (0, 3)	3 (2, 4)	<0.001	2.5 (1, 4)	6 (4, 9)	<0.001
ViEWS	4 (2, 6)	2 (1, 4)	4 (3, 5)	<0.001	3.5 (2, 5)	10 (5, 11)	<0.001

**Abbreviations**: HR, *Heart Rate*; RR, *Respiratory Rate*; SpO2, *Oxygen Saturation*; SBP, *Systolic Blood Pressure*; DBP, *Diastolic Blood Pressure*; MAP, *Mean Arterial Pressure*; GCS, *Glasgow Coma Scale*; Hb, *Haemoglobin*; TLC, *Total Leucocyte Count*, CRP, *C-Reactive Protein*, HTN, *Hypertension*; CAD, *Coronary Artery Disease*; COPD, *Chronic Obstructive Pulmonary Disease*; CKD, *Chronic Kidney Disease*; DM, *Diabetes Mellitus*; Thyroid disease, *Hypothyroidism/Hyperthyroidism*; CNS Disease, *Central Nervous System Disease*; MEWS, *Modified Early Warning Score*; RAPS, *Rapid Acute Physiology Score*; CRB-65, *Confusion Respiratory Rate Blood Pressure Age 65 score*; qSOFA, *quick Sepsis-related Organ Failure Assessment*; SEWS, *Standardized Early Warning Score*; REMS, *Rapid Emergency Medicine Score*; WPS, *Worthing Physiological Scoring system*; NEWS 2, *National Early Warning Score 2*; m-NEWS, *Modified National Early Warning Score*; HEWS, *Hamilton Early Warning Score*; ViEWS, *Vitalpac Early Warning Score*.

^a^Serious illness is defined as a severe or critical COVID-19 case.

^1^Missing value = 49

^2^Missing value = 50

^3^Missing value = 52

^4^Missing value = 51

^5^Missing value = 93

^6^Missing value = 93

^7^Missing value = 110

^8^Missing value = 64

^9^Missing value = 83

Data are presented as median (25^th^, 75^th^ percentile) or n (%)

All values are collected at hospital admission

Among 175 participants having mild to moderate illness, 61 (34.9%) developed a serious illness.

Patients who developed serious illness were comparatively older (56 vs. 42 years, *p* < 0.001), had higher median respiratory rate (28 vs. 20, *p* < 0.001), and lower median diastolic blood pressure (76 vs. 82 mmHg, *p* < 0.004) on admission. Their mean SpO_2_ level was 97% (IQR 96, 98) and none of them required supplemental O_2_ on admission as they developed serious illness later. Patients who developed serious illnesses frequently had a history of HTN (59% vs. 32.5%, *p* < 0.001) and CAD (27.9% vs. 6.1%, *p* < 0.001) (Table 1).

The patients who died tended to be older (65 vs. 49 years, *p* < 0.001). They had a higher median respiratory rate (36 vs. 24, *p* < 0.001), a lower median SpO_2_ (91% vs. 98%, *p* < 0.001) and higher temperature (37.0º vs. 36.3º C, *p* = 0.009) on admission. (Table 1).

The deceased patients were more commonly reported with a history of CAD (40% vs. 12.4%, *p* < 0.001) and CKD (20% vs. 0, *p* <0.001) (Table 1).

The COVID-19 clinical team conducted laboratory investigations depending on the clinical condition and severity of the patients. From the available data, we observed the patients who died had higher total leucocyte count (9.28×10^9^/L vs. 6.71×10^9^/L, *p* = 0.002), differential neutrophil count (83.3% vs. 66.2%, *p* <0.001), creatinine (97.7 μmol/L vs. 73 μmol/L, *p* = 0.001), CRP (15.5 mg/dl vs. 3.55 mg/dl, *p* = 0.008) and D-dimer (755.4 pg/ml vs. 446.6 pg/ml, *p* = 0.003) but lower differential lymphocyte count (11.2% vs. 25.4%, *p* <0.001) than the survivors (Table 1).

Similarly, we observed the patients who developed serious illness had higher differential neutrophil count (69.5% vs. 62.2%, *p* = 0.002) and CRP (4.09 mg/dl vs. 1.49 mg/dl, *p* = 0.004) but lower differential lymphocyte count (23.1% vs. 29.3%, *p* = 0.002) than who did not develop serious illness (Table 1).

All 13 EWSs were evaluated and showed higher scores among those who developed serious illness and died compared to those who did not develop any (Table 1).

### Accuracy of early warning scores in predicting serious illness

Table 2 shows AUROC values, optimal cut-off values, sensitivity, specificity, and accuracy values of all the EWSs chronologically (Table 2).

**Table 2:** Performance of early warning scores in predicting development of serious illness^a^ among COVID-19 positive patients admitted with mild to moderate illness (N = 175).

Score	AUROC	95% CI	Cut-off^b^	Sensitivity (%)	Specificity (%)	Accuracy (%)
m-NEWS	0.766	0.693, 0.839	4	77.1	66.7	70.3
REMS	0.765	0.695, 0.835	3	78.7	58.9	66.0
NEWS2	0.739	0.661, 0.816	4	70.5	71.9	71.4
ViEWS	0.739	0.661, 0.816	4	70.5	71.9	71.4
HEWS	0.737	0.662, 0.813	3	73.8	71.1	72.0
WPS	0.718	0.645, 0.792	2	85.2	47.4	60.6
Groarke	0.715	0.635, 0.790	2	72.1	63.1	66.3
SEWS	0.714	0.638, 0.790	2	65.6	67.5	66.9
MEWS	0.709	0.631, 0.787	3	57.4	78.1	70.9
CRB-65	0.705	0.631, 0.779	1	60.7	77.2	71.4
qSOFA	0.671	0.599, 0.742	1	82.0	48.2	60.0
RAPS	0.666	0.586, 0.746	1	68.8	61.5	64.0
Goodacre	0.651	0.570, 0.733	2	91.8	39.5	57.7

**Abbreviations**: AUROC, *Area Under Receiver Operating Curve*; 95% CI, *95% Confidence Interval*; m-NEWS, *Modified National Early Warning Score*; REMS, *Rapid Emergency Medicine Score*; NEWS 2, *National Early Warning Score 2*; ViEWS, *Vitalpac Early Warning Score*; HEWS, *Hamilton Early Warning Score*; WPS, *Worthing Physiological Scoring system*; SEWS, *Standardized Early Warning Score*; MEWS, *Modified Early Warning Score*; CRB-65, *Confusion Respiratory Rate Blood Pressure Age 65 score*; qSOFA, *quick Sepsis-related Organ Failure Assessment*; RAPS, *Rapid Acute Physiology Score*.

^a^Serious illness is defined as a severe or critical case of COVID-19

^b^Optimal cutoffs were estimated empirically based on highest Youden Index (sum of sensitivity and specificity)

AUROC analysis demonstrated that m-NEWS had the highest AUROC (0.766; 95% CI: 0.693, 0.839). At the optimal cut-off value of 4 or more, it had 77.1% sensitivity, 66.7% specificity, and 70.3% overall accuracy. REMS also showed a good predictive value by showing a good AUROC (0.765; 95% CI: 0.695, 0.835). At an optimal cut-off value 3 or more, it had 78.7% sensitivity, 58.9% specificity with 66.0% accuracy (Table 2).

Among others, NEWS2 and ViEWS both showed the same AUROC’s (0.739; 95% CI: 0.661, 0.816) and HEWS showed an of AUROC 0.737 (95% CI: 0.662, 0.813) in advancing towards development of serious illness (Table 2).

### Accuracy of early warning scores in predicting in-hospital mortality

Among these EWSs, REMS was the most accurate scoring system with highest AUROC (0.89; 95% CI: 0.818, 0.962) among all EWSs for predicting death. At optimal cut-off value 6 or more with 84.0% sensitivity, 77.8% specificity and 78.5% accuracy (Table 3).

**Table 3:** Performance of early warning scores in predicting death among COVID-19 positive patients admitted with mild to serious illness (N = 219).

Score	AUROC	95% CI	Cut-off^b^	Sensitivity (%)	Specificity (%)	Accuracy (%)
REMS	0.890	0.818, 0.962	6	84.0	77.8	78.5
m-NEWS	0.869	0.798, 0.941	6	88.0	69.6	71.7
CRB-65	0.855	0.791, 0.919	1	96.0	62.4	66.2
HEWS	0.841	0.757, 0.925	4	80.0	71.6	72.6
ViEWS	0.824	0.738, 0.909	8	68.0	86.1	84.0
NEWS2	0.820	0.734, 0.905	7	68.0	82.3	80.8
WPS	0.819	0.735, 0.903	3	92.0	64.4	67.6
SEWS	0.818	0.734, 0.902	3	84.0	69.6	71.2
RAPS	0.793	0.705, 0.882	3	72.0	78.4	77.6
Groarke	0.793	0.693, 0.893	4	76.0	73.2	73.5
MEWS	0.749	0.660, 0.839	3	84.0	59.8	62.6
Goodacre	0.717	0.607, 0.827	3	92.0	40.7	46.6
qSOFA	0.656	0.586, 0.726	1	96.0	34.0	41.1

**Abbreviations**: AUROC, *Area Under Receiver Operating Curve*; 95% CI, *95% Confidence Interval*; REMS, *Rapid Emergency Medicine Score*; m-NEWS, *Modified National Early Warning Score*; CRB-65, *Confusion Respiratory Rate Blood Pressure Age 65 score*; HEWS, *Hamilton Early Warning Score*; ViEWS, *Vitalpac Early Warning Score*; NEWS 2, *National Early Warning Score 2*; WPS, *Worthing Physiological Scoring system*; SEWS, *Standardized Early Warning Score*; RAPS, *Rapid Acute Physiology Score*; MEWS, *Modified Early Warning Score*; qSOFA, *quick Sepsis-related Organ Failure Assessment*

^b^Optimal cutoffs were estimated empirically based on highest Youden Index (sum of sensitivity and specificity)

Here m-NEWS stood second-best by showing an AUROC of 0.869 (95% CI: 0.798, 0.941). A cut off value 6 or more demonstrated 88% sensitivity, specificity 69.6% and 71.7% accuracy (Table 3).

Regarding other scores, CRB-65 (AUROC 0.855; 95% CI: 0.791, 0.919), HEWS (AUROC 0.841; 95% CI: 0.757, 0.925), ViEWS (AUROC 0.824; 95% CI: 0.738, 0.909) also proved themselves as good predictors of in-hospital mortality (Table 3).

### Pairwise-comparisons between AUROCs of EWSs with top five AUROCs

In pairwise comparison among the top five AUROC’s of EWSs for predicting serious illness, there was no statistically significant difference between m-NEWS and REMS (*p* = 0.983) (Table 4). Pairwise comparison of top five AUROCs of early warning scores for predicting death similarly showed no significant difference between REMS and m-NEWS (*p* = 0.428) (Table 4).

**Table 4:** Pairwise comparisons of AUROC values of top 5 early warning scores in predicting serious illness^a^ (N = 175) and Death (N = 219).

**Serious Illness**					
**Scores**	**m-NEWS** AUROC = 0.766	**REMS** AUROC = 0.765	**NEWS2** AUROC = 0.739	**ViEWS** AUROC = 0.739	**HEWS** AUROC = 0.737
**m-NEWS** AUROC = 0.766	-	0.983	0.185	0.185	0.260
**REMS** AUROC = 0.765	0.983	-	0.540	0.540	0.495
**NEWS2** AUROC = 0.739	0.185	0.540	-	-	0.947
**ViEWS** AUROC = 0.739	0.185	0.540	-	-	0.645
**HEWS** AUROC = 0.737	0.260	0.495	0.947	0.645	-
**Death**					
**Scores**	**REMS** AUROC = 0.890	**m-NEWS** AUROC = 0.869	**CRB-65** AUROC = 0.855	**HEWS** AUROC = 0.841	**ViEWS** AUROC = 0.824
**REMS** AUROC = 0.890	-	0.428	0.347	0.233	0.098
**m-NEWS** AUROC = 0.869	0.428	-	0.678	0.208	0.041
**CRB-65** AUROC = 0.855	0.347	0.678	-	0.766	0.507
**HEWS** AUROC = 0.841	0.347	0.061	0.979	-	0.934
**ViEWS** AUROC = 0.824	0.098	0.041	0.507	0.366	-

**Abbreviations**: AUROC, *Area Under Receiver Operating Curve*; m-NEWS, *Modified National Early Warning Score*; REMS, *Rapid Emergency Medicine Score*; NEWS 2, *National Early Warning Score 2*; ViEWS, *Vitalpac Early Warning Score*; HEWS, *Hamilton Early Warning Score*; CRB-65, *Confusion Respiratory Rate Blood Pressure Age 65 score*.

^a^Serious illness is defined as a severe or critical case of COVID-19.

Cell values are *p* values obtained from DeLong tests comparing the equality of the AUROCs.

## Discussion

Currently, no EWS is in use in our hospital while not mentioning other hospitals in Bangladesh for the screening of COVID-19 patients. To the best of our knowledge, this is the first study in Bangladesh that has assessed the predictive value of EWSs in predicting outcomes in adult COVID-19 patients.

The median age of 219 COVID-19 patients was 51 (38, 60) years. We found that the proportion of severe or critical COVID-19 and in-hospital mortality was significantly higher among older individuals. The study from Chongqing, China [38] also showed that age is an independent risk factor for severe COVID-19. We observed in our study, deceased patients had high level of total leucocytes count, neutrophil, CRP, D-Dimer, and low lymphocyte than the survivors. A study done in similar set up in Bangladesh during COVID-19 era, the study team found that the critically ill patients had lymphopenia, high D-Dimer and CRP level than those who are not critically ill [39].

**Fig 2:**
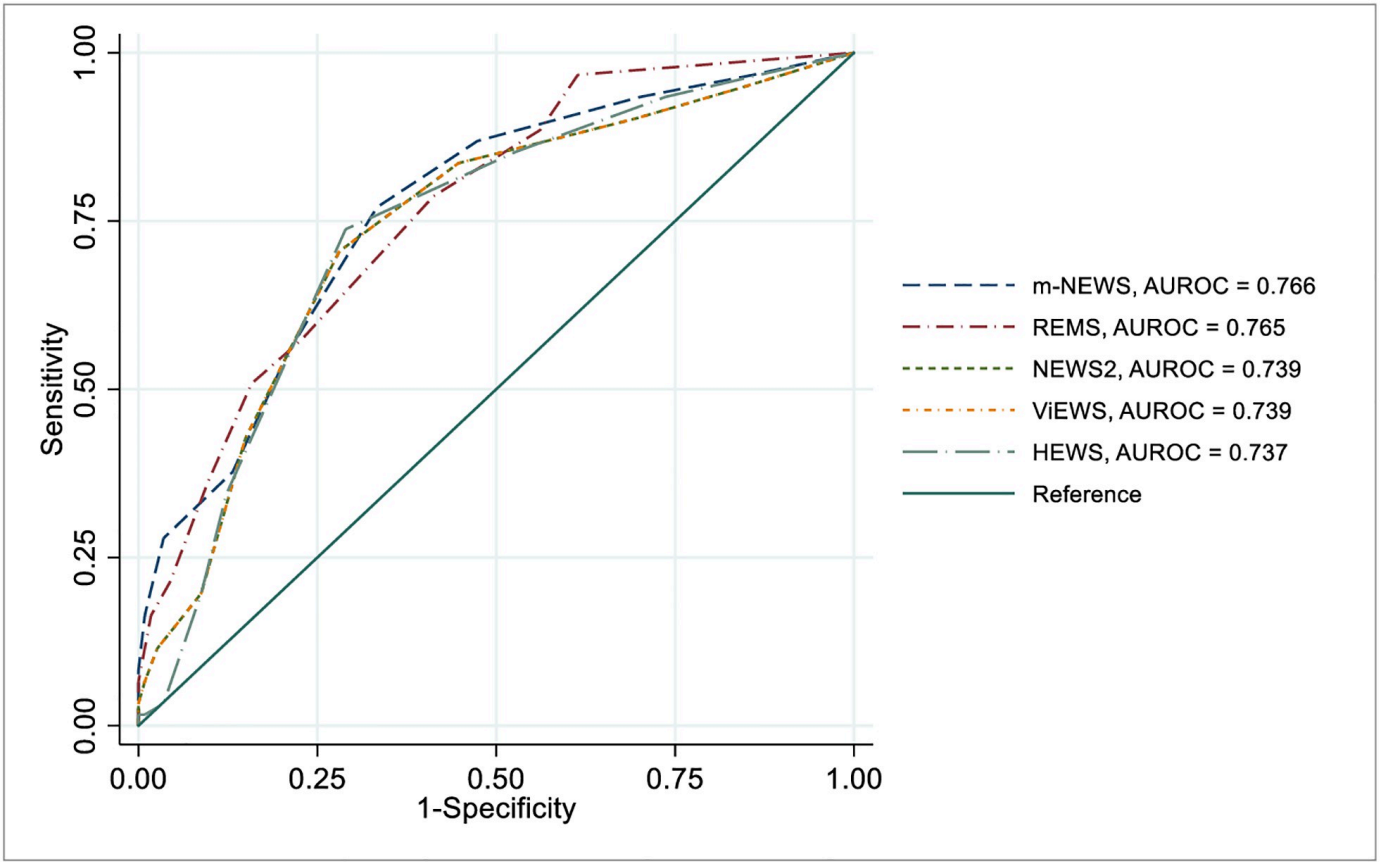
Receiver operating characteristic (ROC) curves of early warning scores for predicting serious illness. Serious illness is defined as a severe or critical COVID-19 case. ROC curves with top 5 AUROCs are displayed. **Abbreviations**: AUROC, *Area Under Receiver Operating Curve*; m-NEWS, *Modified National Early Warning Score*; REMS, *Rapid Emergency Medicine Score*; NEWS 2, *National Early Warning Score 2*; ViEWS, *Vitalpac Early Warning Score*; HEWS, *Hamilton Early Warning Score*.

**Fig 3:**
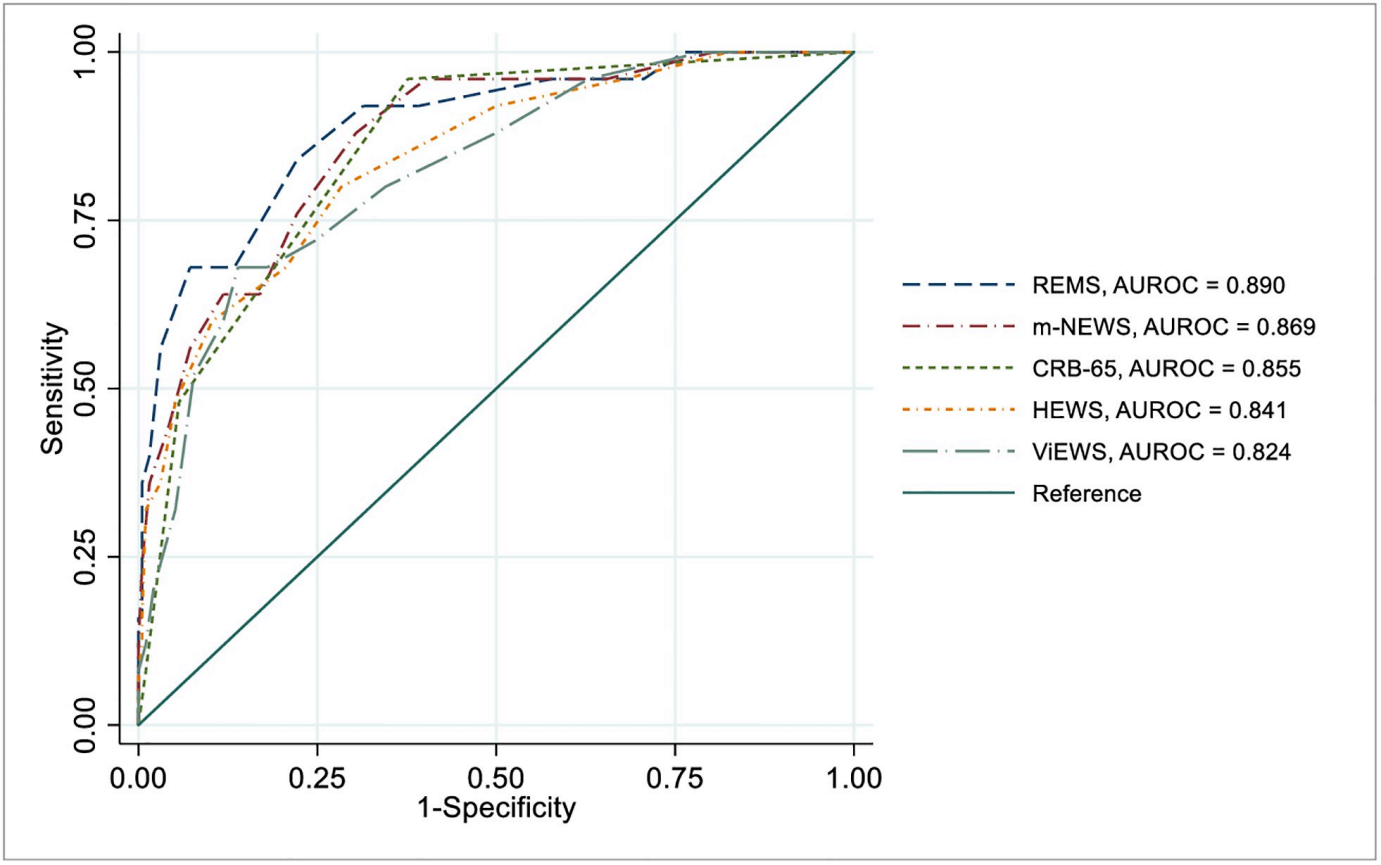
Receiver operating characteristic (ROC) curves of early warning scores for predicting death. ROC curves with top 5 AUROCs are displayed. **Abbreviations**: AUROC, *Area Under Receiver Operating Curve*; REMS, *Rapid Emergency Medicine Score*; m-NEWS, *Modified National Early Warning Score*; CRB-65, *Confusion Respiratory Rate Blood Pressure Age 65 score*; HEWS, *Hamilton Early Warning Score;* VIEWS, *Vitalpac Early Warning Score*.

Our study showed that m-NEWS and REMS assessed on admission demonstrated the highest AUROC point estimates for predicting serious illness and in-hospital mortality in COVID-19 patients, respectively. Modified NEWS termed as m-NEWS or NEWS-C showed best predictive value by representing excellent AUROC 0.766 for developing serious illness among all other scores (Fig 2) and performed second best with good AUROC 0.869 regarding the prediction of death (Fig 3). The finding is similar to the study performed in Zhongshan Hospital, where they found m-NEWS with the largest AUROC value for predicting early deterioration of respiratory function (EDRF) and any need for intensive respiratory support (IRS) [11]. This score already suggested in China for making triage decisions for patients with COVID-19 and we found the nearest cut-off value like them for predicting death [25]. The retrospective study performed in Alzano Lombardo hospital, Italy, indicated m-NEWS score 7 or greater at admission was associated with a negative outcome, which means patients need more intensive care and treatment, whereas patients who had m-NEWS score on admission under 7 were associated with a higher chance of recovery [6]. In the case of anticipating serious illness m-NEWS had less cut-off value, which is 4 or more might be the reason for excluding patients who had a serious illness during admission time as we want to prognosticate the patients who will get severe or critical COVID-19 later. m-NEWS is the modified version of the NEWS score by adding the age variable ≥65 years, making it a stronger one for assessing COVID-19 patients [6]. Multiple studies have shown that older age was independently associated with death COVID-19 patients [11, 40]. In this scoring system, patients are stratified into 5 warning levels with different colours on chart. More the score darker the colours; white (0), yellow (1–4), orange (5–6), red and black (≥ 7) [6, 25]. Recently a study in France found m-NEWS not to be a good performer in predicting ICU admission or death [41]. However, it is already recommended in China and Italy for use in COVID-19 patients [6, 25].

Rapid Emergency Medicine Score (REMS) is the modified version of RAPS which was originated from Acute Physiology and Chronic Health Evaluation II (APACHE II) to predict in hospital mortality of patients [31]. This score was first validated in 2003 which predicted good in hospital mortality among non-surgical patients [30]. It was modified by adding oxygen saturation and age to other variables in the scoring system. In our study, for predicting in-hospital mortality, REMS (AUROC 0.890) was the most accurate scoring system of all EWSs. In predicting serious illness development, REMS took second place. The study performed on 392 patients admitted to the emergency department in a hospital in Turkey displayed REMS as a useful scoring system in predicting 1-month mortality and clinical outcome [42]. A study from the USA on trauma population and another study in the UK in the emergency department showed REMS performed better as a predictor of mortality in emergency medical admissions than other scores [31, 43].

The NEWS 2 is the updated version of NEWS and it is now suggested by the Royal College of Physicians to predict clinical deterioration in patients with COVID-19 [44]. It includes a new SpO2 scoring scale for type 2 respiratory failure patients to avoid unnecessary use of supplemental oxygen and to assist the clinicians to better manage these patients. In our study, NEWS2 performed lower than m-NEWS in predicting serious illness that is severe and critical COVID-19 patients. It may be because there was no patient of COVID-19 with type II respiratory failure in our population, similar to data reported by a Chinese study from Wuhan and an Italian study [11, 44]. Data from another study showed that hypercapnia is not very common among COVID-19 patients, even in those who needed admission in ICU [45].

We observed in our study that those scores that do not consider supplemental oxygen and SpO_2_ level performed worst in predicting both outcomes that are serious illness and death. This finding has similarity with the study undertaken in China, where MEWS performed worst in predicting mortality which does not require SpO_2_ or supplemental O_2_ for calculating score [9]. In our study, CRB-65 performed well in predicting death, while on the contrary, information gathered from a Chinese study done in 2020 revealed CRB-65 score as a poor predictor of death in COVID-19 patients [46]. However, in our study, CRB-65 score gave a poor performance in predicting serious illness. The qSOFA score displayed a very good sensitivity (96%) like CRB-65 for predicting death in ED patients but was less accurate (41.1%) than other early warning scores. Churpek et al. found that other general early warning scores used in their study were more accurate than qSOFA as a predictor marker for mortality in patients with suspected infection [47]. Our results indicate that scores who do not include SpO_2_ or O_2_ therapy for calculating scores may not be an effective tool for diagnosing severe and critically ill patients with COVID-19.

Additionally, ViEWS and HEWS showed good discriminative ability among 13 EWSs overall and the subgroup analysis in predicting both serious illness and death. A study in the UK [16] reported that ViEWS’s performance was better than other scores in the deterioration of adult inpatients. A report from China [9] demonstrated HEWS as a better score to screen critical COVID-19 patients.

Both m-NEWS and REMS showed their potential to predict the development of serious illness and death. REMS has not been widely studied for use in predicting outcomes of COVID-19 patients, but recently, a study carried out in the USA exhibited pre-hospital calculation of REMS score can predict outcome in COVID-19 patients [48]. m-NEWS is already recommended for risk stratification of COVID-19 patients on admission in a few countries where a doctor might get a chance to visit patients once due to the overburden of patients. Even in the absence of a clinician, any healthcare provider can calculate the score, which will eventually bring down the worsening of the patients in the future [6, 25, 49]. Since the beginning of the year, COVID-19 activity has increased due to the ongoing global rise in the prevalence of a new variant. Countries all over the world are putting new laws into place to slow the spread of respiratory illnesses like COVID-19, like putting temperature monitors in airports and encouraging people to wear masks again. To estimate the disease’s severity and stop premature death, a simple bedside scoring system like m-NEWS is required especially in low-resource countries like ours.

### Limitations

Despite being the first study to explore the accuracy of early warning scoring systems for patients with COVID-19 in Bangladesh, our study still has some limitations. First, it was a single-center study with a limited number of participants therefore, the results may not be generalizable to other settings. Second, we obtained the data retrospectively from the patients’ medical records, which was not collected specifically for research purposes. Third, it was done in a specific group of the population, that is, the staff of this institution and their family members. Fourth, we did not have data about receiving O_2_ supplementation before admission, which could affect the calculation of the total score in emergency department arrival. Fifth, we did not take into consideration the role of COVID-19 variant differentials in disease severity and case fatality as observed in different countries of the world. Therefore, a prospective multi-centre study with larger sample size will aid in providing an even more accurate assessment of EWS in predicting adverse outcomes in COVID-19 patients.

## Conclusion

In our study, m-NEWS and REMS performed best with fair AUROC’s for predicting serious illness and in-hospital mortality, respectively, at admission. However, we did not find any significant difference between m-NEWS and REMS and some other EWSs, such as NEWS2, ViEWS, HEWS, etc. in terms of AUROCs. Scores that do not account for SpO2 or O2 therapy in their calculation may not be useful in identifying severe COVID-19 cases. We propose m-NEWS could be used as a triage score to identify COVID-19 patients at risk of disease progression and death, especially in resource-poor settings because it has been explicitly developed for risk stratification of COVID-19 patients in some countries like China and Italy. However, this tool needs to be validated by further large-scale prospective studies.

## Supporting information

S1 Table Early warning scores considering their use in different emergency settings and outcomes by SpO_2_ and supplemental O_2_ for calculation [6, 10, 14, 16, 21, 23–25, 27, 29, 30–32, 34, 50–54]. (PDF)

S2 Table Early warning scores with the scoring charts. (PDF)
