# Supplementary material for: Prognostic accuracy of early warning scores for predicting serious illness and in-hospital mortality in patients with COVID-19
Source: PLOS Glob Public Health. 2024 Mar 28;4(3):e0002438. doi: 10.1371/journal.pgph.0002438 (PMC10977747; doi:10.1371/journal.pgph.0002438)
Supplement: S1 Table — (PDF) [file pgph.0002438.s001.pdf]

S1 Table. Early warning scores considering their use in different emergency settings and outcomes by SpO_2 and supplemental O_2 for calculation

Categories	EWS	Derived Population	Predicted Outcomes
EWSs that do not require SpO_2 or supplemental O_2 to calculate the total score	MEWS [10,14]	Medical emergency admission patients	Admission to ICU or HDU, cardiac arrest, survival and discharge at 60 days, in hospital mortality
	RAPS [14,27]	Critical care pre-hospital transport patients	In hospital mortality
	Goodacre [14,31]	Patients in ED transported by ambulance	In hospital mortality
	CRB-65 [50]	Both in hospital and out-patients setting to assess community acquired pneumonia (CAP)	Death from CAP in both hospitalized and out-patients
	qSOFA [51]	Patients with suspected infection on pre-hospital or emergency admission	Prediction of mortality in critically ill septic patients in prehospital, emergency department and ward setting
EWSs that consider SpO_2 but not supplemental O_2 to calculate the total score	SEWS [29,52]	Emergency medical admissions	In hospital mortality and length of stay
	REMS [14,30]	Patients in ED	Length of stay, in hospital mortality
	Groarke [14,34]	Medical admission patients	Admission to ICU, cardiac arrest, length of stay, in hospital mortality

	WPS [14,32]	Patients in ED	In hospital mortality
EWSs that consider both SpO_2 and requirement of supplemental O_2 to calculate the total score	NEWS2 [23,24,53]	Acute medical admission patients, hypercapnic (often termed type 2) respiratory failure, serious sepsis in patients, COVID-19 patients	Admission to ICU, cardiac arrest, 24-hour mortality, clinical deterioration due to COVID-19
	m-NEWS [6,25]	Acute medical admission of COVID-19 patients	Death and ICU admission due to COVID-19
	HEWS [54]	Medical admission patients	Admission to ICU, cardiac arrest, in hospital mortality
	ViEWS [14,16,21]	Acute medical admission patients	24-hour mortality on admission
