# Supplementary material for: Prognostic accuracy of early warning scores for predicting serious illness and in-hospital mortality in patients with COVID-19
Source: PLOS Glob Public Health. 2024 Mar 28;4(3):e0002438. doi: 10.1371/journal.pgph.0002438 (PMC10977747; doi:10.1371/journal.pgph.0002438)
Supplement: S2 Table — (PDF) [file pgph.0002438.s002.pdf]

S2 Table. Early warning scores with the scoring charts

NEWS2

**Physiological Parameters**	3	2	1	0	1	2	3
RR	≤8		9-11	12-20		21-24	≥25
SpO_2 scale 1 (%)	≤91	92-93	94-95	≥96			
SpO_2 scale 2 (%)	≤83	84-85	86-87	88-92 ≥93 on air	93-94 on oxygen	95-96 on oxygen	≥97 on oxygen
Air or Oxygen		Oxygen		Air			
SBP (mmHg)	≤90	91-100	101-110	111-219			≥220
Pulse (Per Minute)	≤40		41-50	51-90	91-110	111-130	≥131
Consciousness				Alert			CVPU
Temperature	≤35		35.1-36.0	36.1-38.0	38.1-39.0	≥39.1	

m-NEWS

**Parameters**	**3**	**2**	**1**	**0**	**1**	**2**	**3**
Age				<65			≥65
Respiratory Rate	≤8		9-11	12-20		21-24	≥25
O2 saturation	≤91	92-93	94-95	≥96			
Supplemental O2		Yes		No			
Systolic Blood Pressure	≤90	91-100	101-110	111-219			≥220
Heart Rate	≤40		41-50	51-90	91-110	111-130	≥131
Consciousness				Alert			Drowsiness Lethargy Coma Confusion
Temperature	≤35		35.1-36	36.1-38	38.1-39	≥39.1	

qSOFA

**Physiological Parameters**	Value
Altered mental status	GCS<15
Tachypnoea	RR ≥22
Hypotension	SBP ≤100mmHg

RAPS

**Variables**	**+4**	**+3**	**+2**	**+1**	**0**	**+1**	**+2**	**+3**	**+4**
MAP	≥160	130-159	110-129		70-109		50-69		≤49
HR	≥180	140-179	110-139		70-109		55-69	40-54	≤39
RR	≥50	35-49		25-34	12-24	10-11	6-9		≤5
GCS					≥14	11-13	8-10	5-7	≤4

MEWS

**Parameters**	**3**	**2**	**1**	**0**	**1**	**2**	**3**
Respiratory Rate		<9		9-14	15-20	21-29	≥30
Heart Rate		≤40	41-50	51-100	101-110	111-129	≥130
Systolic Blood Pressure	≤70	71-80	81-100	101-199		≥200	
Temperature		<35		35-38.4		≥38.5	
Consciousness				Alert	Voice	Pain	Unresponsive

SEWS

**Parameters**	**3**	**2**	**1**	**0**	**1**	**2**	**3**
Respiratory Rate	<9			9-20	21-30	31-35	>35
SpO_2	<85	85-89	90-92	93-100			
Temperature	<34	34-34.9	35-35.9	36-37.9	38-38.9	>38.9	
Systolic Blood Pressure	<70	71-79	80-99	100-199		>199	
Heart Rate	<30	30-39	40-49	50-99	100-109	110-129	>129
Consciousness				A	V	P	U

REMS

**Variable/ score**	**+4**	**+3**	**+2**	**+1**	**0**	**1**	**2**	**3**	**4**	**5**	**6**
MAP	>159	130-159	110-129		70-109		50-69		≤49		
HR	>179	140-179	110-139		70-109		55-69	40-54	<39		
RR	>49	35-49		25-34	12-24	10-11	6-9		<5		

SaO_2					>89	86-89		75-85	<75		
GCS					>13	11-13	8-10	5-7	3-4		
Age					<45		45-54	55-64		65-74	>74

Goodacre

**Variable/ score**	**0**	**+1**	**+2**	**+3**	**+4**	**+5**	**+6**
GCS	>13	11-13	8-10	5-7	<5		
HR	>89	86-89		75-85	<75		
Age	<45		45-54	55-64		65-74	>74

WPS

**Variables**	**0**	**+1**	**+2**	**+3**
RR	≤ 19	20-21	≥22	
HR	≤ 101	≥ 102		
SBP	≥ 100		≤99	
T	≥35.3			<35.3
SaO_2	96-100≥	94 - 95	92-93	<92
AVPU	A			V, P or U

HEWS

**Variables**	**3**	**2**	**1**	**0**	**1**	**2**	**3**
HR		<40	41-50	51-100	101-110	111-130	>130
SBP	<70	71-90				171-200	>200
RR	<8	8-13				21-30	>30
T	<35		35.1-36.4	36.5-38	38.1- 39	>39.1	
O_2 saturation	<85		85-91	>92			
Oxygen delivery				Room air	≤5L/ min or ≤50% by mask		>5L/ min or >50% by mask
Neurologic status		CAM positive		Alert	Voice	Pain	Unresponsive

Groarke

**Variables**	**+3**	**+2**	**+1**	**0**	**1**	**2**	**3**
HR	≤40	41-50		51-100	101-110	111-129	>130
SBP	≤80	81-90	91-100	101-199		>200	
RR	≤8			9-19	20-25	25-29	>30
T		≤35.0		35.1-38		38.1-39.5	>39.5

Consciousness				A	V	P	U
SaO_2	<85%	85-89	90-94	>95%			

ViEWS

**Variables**	**+3**	**+2**	**+1**	**0**	**+1**	**+2**	**+3**
RR	≤8		9-11	12-20		21-24	≥25
SaO_2	≤91	92-93	94-95	≥96			
Supplemental O_2	Yes			No			
T	≤35.0		35.1-36	36.1-38	38.1-39	≥39.1	
SBP	≤90	91-100	101-110	111-249	≥250		
HR		≤40	41-50	51-90	91-110	111-130	≥131
AVPU				A			V, P, U

CRB-65

**Clinical Factor**	**Points**
Confusion	1
RR ≥30 breaths/minute	1
SBP <90 mmHg or DBP ≤ 60 mmHg	1
Age ≥ 65 years	1

Abbreviations: HR, *Heart Rate*; RR, *Respiratory Rate*; SpO2, *Oxygen Saturation*; SBP, *Systolic Blood Pressure*; T, *Temperature*; SaO_2, *Arterial Oxygen Saturation;* DBP, *Diastolic Blood Pressure*; MAP, *Mean Arterial Pressure*; GCS, *Glasgow Coma Scale*; AVPU, *Alertness, Response to Voice and Pain, Unresponsive scale*
